# Minimum detectable spinal cord atrophy with automatic segmentation: Investigations using an open-access dataset of healthy participants

**DOI:** 10.1016/j.nicl.2021.102849

**Published:** 2021-10-04

**Authors:** Paul Bautin, Julien Cohen-Adad

**Affiliations:** a NeuroPoly Lab, Institute of Biomedical Engineering, Polytechnique Montreal, Montreal, QC, Canada; b Functional Neuroimaging Unit, CRIUGM, Université de Montréal, Montreal, QC, Canada; c Mila - Quebec AI Institute, Montreal, QC, Canada

**Keywords:** ALS, Amyotrophic Lateral Sclerosis, CSA, Cross-Sectional Area, CSF, Cerebrospinal Fluid, MS, Multiple Sclerosis, NMOSD, Neuromyelitis Optica Spectrum Disorder, SD, Standard Deviation, SC, Spinal Cord, SCT, Spinal Cord Toolbox, PVE, Partial Volume Effect, SI, Superior-Inferior, Atrophy, Simulation, Spinal cord, Sample size

## Abstract

Spinal cord atrophy is a well-known biomarker in multiple sclerosis (MS) and other diseases. It is measured by segmenting the spinal cord on an MRI image and computing the average cross-sectional area (CSA) over a few slices. Introduced about 25 years ago, this procedure is highly sensitive to the quality of the segmentation and is prone to rater-bias. Recently, fully-automated spinal cord segmentation methods, which remove the rater-bias and enable the automated analysis of large populations, have been introduced. A lingering question related to these automated methods is: How reliable are they at detecting atrophy? In this study, we evaluated the precision and accuracy of automated atrophy measurements by simulating scan-rescan experiments.

Spinal cord MRI data from the open-access spine-generic project were used. The dataset aggregates 42 sites worldwide and consists of 260 healthy subjects and includes T1w and T2w contrasts. To simulate atrophy, each volume was globally rescaled at various scaling factors. Moreover, to simulate patient repositioning, random rigid transformations were applied. Using the DeepSeg algorithm from the Spinal Cord Toolbox, the spinal cord was segmented and vertebral levels were identified. Then, the average CSA between C3-C5 vertebral levels was computed for each Monte Carlo sample, allowing us to derive measures of atrophy, intra/inter-subject variability, and sample-size calculations.

The minimum sample size required to detect an atrophy of 2% between unpaired study arms, commonly seen in MS studies, was 467 +/− 13.9 using T1w and 467 +/− 3.2 using T2w images. The minimum sample size to detect a longitudinal atrophy (between paired study arms) of 0.8% was 60 +/− 25.1 using T1w and 10 +/− 1.2 using T2w images. At the intra-subject level, the estimated CSA, observed in this study, showed good precision compared to other studies with COVs (across Monte Carlo transformations) of 0.8% for T1w and 0.6% for T2w images.

While these sample sizes seem small, we would like to stress that these results correspond to a “best case” scenario, in that the dataset used here was of particularly good quality and the model for simulating atrophy does not encompass all the variability met in real-life datasets. The simulated atrophy and scan-rescan variability may over-simplify the biological reality. The proposed framework is open-source and available at https://csa-atrophy.readthedocs.io/.

## Highlights

• Evaluate the robustness of an automated analysis pipeline for detecting SC atrophy.
• Simulate spinal cord atrophy and scan-rescan variability.
• Fully automated analysis method available on an open access database.
• Evaluation of sample size and inter/intra-subject variability for T1w and T2w images.

## 1 Introduction

### 1.1 Spinal cord atrophy, description and causes

Spinal cord (SC) atrophy is characterized by the progressive loss of SC parenchyma and can occur in a variety of diseases, including Multiple Sclerosis (MS) (Trapp and Nave, 2008), Amyotrophic Lateral Sclerosis (ALS) (Wimmer et al., 2020), Neuromyelitis Optica Spectrum Disorder (NMOSD) (Lersy et al., 2021), Alzheimer’s disease (Lorenzi et al., 2020) and traumatic injuries (Ziegler et al., 2018). In MS, distinct phenotypes are associated with different SC atrophy rates; thus it is a relevant biomarker for diagnosis and prognosis (Moccia et al., 2019, van Faals et al., 2020). Precise and accurate monitoring of SC atrophy over time offers high prognosis value (Sastre-Garriga et al., 2020). Pooled annual atrophy rates, found in Casserly et al. *meta*-study (Casserly et al., 2018), were 1.78% per year for all types of MS (mean rate across 22 studies) and 2.08% per year for progressive MS (mean rate across 15 studies). Typical atrophy rates for different pathologies are presented in supplementary material **Table S1**.

### 1.2 How to measure SC atrophy?

SC atrophy is typically measured by segmenting the SC on an MRI image and computing its CSA (Losseff et al., 1996). The precision of CSA is primarily limited by the axial image resolution (Tardif et al., 2009), therefore averaging the CSA over multiple slices increases this precision. To minimize rater bias, several segmentation methods have been developed over the past three decades, with varying degrees of automation (De Leener et al., 2016, Weeda et al., 2019). Notably, a study by Yiannakas et al. (Yiannakas et al., 2016) found good agreement between a semi-automatic (Horsfield et al., 2010) and the fully automatic PropSeg (De Leener et al., 2014) segmentation method available in SCT. Other studies have also used SCT to assess SC atrophy in MS (Combes et al., 2017, Mariano et al., 2021, Weeda et al., 2019), amyotrophic lateral sclerosis (Paquin et al., 2018, Querin et al., 2021), spinal muscular atrophy (Querin et al., 2019), neuromyelitis optica spectrum disorders (Lersy et al., 2021, Mariano et al., 2021, Ventura et al., 2016), degenerative cervical myelopathy (Martin et al., 2018, Ost et al., 2021), traumatic SC injury (Azzarito et al., 2020), adrenomyeloneuropathy (Adanyeguh et al., 2021) and MOG-antibody disease (Mariano et al., 2021), including longitudinal studies looking at atrophy change over time (Combes et al., 2017, Martin et al., 2018, Querin et al., 2021).

When comparing absolute CSA across groups, one is faced with the relatively large variation of SC morphometry across individuals. For example, Yiannakas et al. reported an inter-subject CSA standard deviation of 7.1 mm^2^ (9.81%) (Yiannakas et al., 2016), which is large compared to an expected atrophy rate of ∼ 2%. The typical procedure for assessing atrophy over time is to repeat an MRI scan and to compute CSA at each time point (Lin et al., 2003, Weeda et al., 2019, Zivadinov et al., 2008). This procedure is hampered by scan-rescan variability (e.g., subject repositioning, motion artifacts, and noise) and by the reproducibility of the image analysis pipeline especially during image segmentation. The accumulation of these errors, when performed across several time points, can significantly hinder the detection sensitivity of subtle atrophy rates. Prados et al. have addressed this problem by using a generalized boundary shift integral (GBSI) method, which computes atrophy measures after co-registering data across time points (Freeborough and Fox, 1997). While this approach bypasses the above stated error accumulation, it remains sensitive to the quality of the co-registration. The outcome of these developments highlights the pertinence of quantifying the sensitivity of state-of-the-art methods for measuring atrophy rates.

In this study, we evaluate the robustness and the sensitivity of an automated analysis pipeline for detecting SC atrophy. To perform this evaluation, a realistic simulation framework was developed following similar approaches to those previously used in the brain (Bernal et al., 2021, Boyes et al., 2006, Camara et al., 2006, Karaçali and Davatzikos, 2006, Khanal et al., 2017). Notably, the proposed framework utilizes image scaling and applies a random rigid transformation to mimic subject repositioning (scan-rescan) enabling the quantification of the accuracy and precision of the estimated CSA across various degrees of simulated atrophy. From these experiments, power analyses and minimum sample sizes are derived. Our simulations are based on an open-access multi-center and multi-vendor (GE, Philips, Siemens) database of 260 subjects (Cohen-Adad et al., 2021).

## 2 Methods

### 2.1 Data

We used data from the spine-generic multi-subject database (Cohen-Adad et al., 2021) version r20201130^1^. This repository contains MRI data from 260 healthy participants with multiple contrasts including T1-weighted (T1w) and T2w which are used in this study. The vendor-specific sequences used were: BRAVO/IR-FSPGR (GE), T1TFE (Philips), MPRAGE (Siemens) for T1w images and CUBE (GE), VISTA (Philips), SPACE (Siemens) for T2w images. For details of the protocol, please refer to https://github.com/spine-generic/protocols. For confidentiality reasons, the faces of subjects were removed (defaced). Particularly useful, this database follows the BIDS convention (Gorgolewski et al., 2016), making the analysis framework developed here compatible with any other BIDS dataset.

### 2.2 Processing

**Fig. 1:**
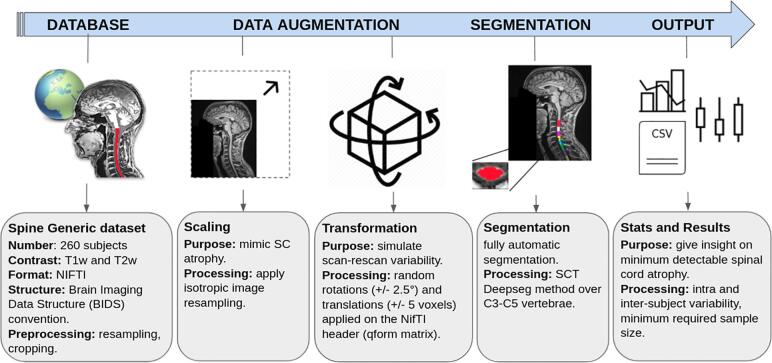
Csa-atrophy pipeline.

Processing code was done using Python 3.7, and the script specific to this study is available as open-source (https://github.com/sct-pipeline/csa-atrophy). Dependent software package, including SCT v5.1.0 (De Leener et al., 2017a) was used. Fig. 1 shows an overview of the processing and evaluation pipeline.

#### 2.2.1 Image scaling to simulate atrophy

**Fig. 2:**
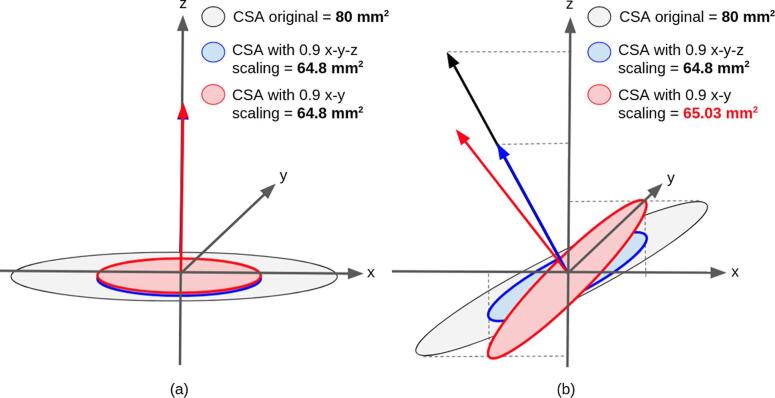
Impact of anisotropic scaling on the estimated CSA. In this example the SC is represented by a disc of radius $R=\sqrt{\frac{80}{\pi }}$ mm, yielding an area of 80 mm^2^. **(a)** In a scenario where the SC centerline is collinear to the vector normal to the axial slice (z), the CSA is scaled by the same factor whether the scaling is isotropic (x-y-z) or only along x-y. In this example, a scaling of 0.9 in each axis yields 80×(0.9)^2^ = 64.8 mm^2^. **(b)** In a scenario where there is an angle between the cord centerline and the vector normal to the axial slice (z), the CSA submitted to an isotropic scaling is independent from that angle, and the scaled CSA is the same as in (a): $\mathrm{CS}A_{S_{x-y-z}}=80\times {\left(0.9\right)}^{2}=64.8\;mm^{2}$. However, the x-y scaling does create a dependency on that centerline angle. For example, with a 10° angle in the (x,z) plane between the SC centerline and the vector normal to the axial slice (z), only the projection on the (x,y) plane is scaled. The scaled projection of the radius R on the x-axis is $Rs_{x}=0.9\times cos\left(10\right)\times R$, while on the y-axis it is $R_{s_{Y}}=0.9\times R$, and on the z-axis it is $R_{s_{z}}=sin\left(10\right)\times R$ (no scaling along z). Using the formula for the area of an ellipse $CSA_{S_{x,y}}=\pi \times R_{S_{x,z}}\times R_{s_{y}}$ and the pythagorean theorem $Rs_{x,z}=\sqrt{{{Rs}_{x}}^{2}+{{Rs}_{z}}^{2}}$we find:$CSA_{S_{x-y}}=\pi \times R_{S_{x,z}}\times R_{s_{y}}=\pi \times sqrt\left[{\left(0.9\times cos(10)\times R\right)}^{2}+{\left(sin(10)\times R\right)}^{2}\right]\times 0.9\times R=65.03\;mm^{2}$.

Prior to processing, all images were resampled to 1 mm isotropic (T1w) and 0.8 mm isotropic (T2w). To mimic SC atrophy a global scaling was applied on each image using a homothetic transformation, on all three axes (x,y,z), in order to preserve the global morphometry (shape) of the SC. While a x-y scaling would appear to be more realistic from the standpoint of cord atrophy (because tissue atrophy mostly occurs in the antero-posterior and right-left axes), there is a major flaw associated with this approach: x-y scaling is only valid if the spinal cord centerline is perfectly orthogonal to the axial slice. If it is not the case, the x-y scaling would produce a non-linear deformation (dependent on the SC morphometry), introducing a dependency of the estimated CSA on the angle between the centerline and the axial slice. This phenomenon is illustrated in Fig. 2. We thus opted for an isotropic scaling.

In order to simulate real-world atrophy studies in which patients (becoming atrophic over time) are followed up in different visits the scaling factor is combined with the affine transformation matrices (see *2.2.2. Transformation*). This scaling factor was then used to compare the estimated SC atrophy versus the *true* atrophy (simulated, with known scaling factor). The idea of combining the scaling factor and the affine transformation matrix is to only do one image resampling (instead of two) and thus minimize interpolation errors.

#### 2.2.2 Transformation

Clinical trials often rely on longitudinal studies to measure atrophy progression. This approach naturally comes with a scan-rescan variability partly caused by repositioning of the subject in the scanner. To mimic this variability, 30 uniformly distributed random 3D rotations (+/- 2.5°) and 3D translations (+/- 5 voxels) were combined with the scaling matrix obtained in *2.2.1.*, and applied on each subject image. This resulted in 30 Monte Carlo samples per subject and per scaling. The data was then resampled using 5th order sinc interpolation. Once completed, all transformations were stored in a CSV file so that results could be reproduced by using the frozen parameters in subsequent runs of the pipeline.

#### 2.2.3 Segmentation

SC segmentation was done using SCT’s sct_deepseg_sc, which is based on the DeepSeg algorithm (Gros et al., 2019). This method consists in finding the SC centerline using a support vector machine combined with histogram oriented gradients algorithm (SVM-HOG), called the “OptiC” method (Gros et al., 2018), followed by a cropping around the centerline and segmentation using a Convolutional Neural Network (CNN), with a 2D kernel. DeepSeg was trained on images with SC pathologies (MS, ALS, compression), and included scaling in data augmentation; hence its performance is robust with regard to SC pathologies and atrophy.

#### 2.2.4 Vertebral labeling

Vertebral labeling was performed using SCT’s sct_label_vertebrae. In brief, the disc C2-C3 is identified using the OptiC algorithm, then the other intervertebral discs are found using SC straightening (De Leener et al., 2017b) followed by template matching (Ullmann et al., 2014) with the PAM50 template (De Leener et al., 2018). Following the identification of the discs, the SC segmentation produced above is labeled with the respective vertebral levels. In cases where automatic labeling failed, the problematic subjects were manually-labeled by an expert and uploaded to the spine-generic database, as detailed in the spine-generic documentation^2^.

#### 2.2.5 Computing CSA

CSA was computed using SCT’s sct_process_segmentation, which sums the number of pixels for each axial slice and multiplies them by the pixel area. The estimated CSA is then corrected slice-wise using the cosine of the angle between the axial plane and the SC centerline (regularized using spline functions). The CSA was then averaged between vertebral levels C3 and C5 (included). The number of slices yielding this coverage was 49.7+/- 4.7 for T1w and 61.9 +/− 5.7 for T2w (across all subjects). The reason for the higher number of slices for T2w is due to the smaller voxel size (0.8 mm vs. 1 mm for T1w).

### 2.3 Statistics

We denote $\mathrm{CS}A_{\mathrm{sI},rX,tY}$ the CSA computed for subject *sI*, scaling factor *rX* and transformation *tY*. The first metrics of interest are the intra-subject CSA variability, which is represented by the standard deviation across transformations: $\sigma_{t}\left\{CSA_{\mathrm{sI},rX}\right\}$ and the coefficient of variation: ${\mathrm{COV}}_{t}\left\{CSA_{\mathrm{sI},rX}\right\}$. These metrics aim at representing a scan-rescan variability, although without the additional “real-life” factors contributing to scan-rescan variance such as different shimming parameters, scanner drifts and motion patterns. These intra-subject metrics were then averaged across subjects, yielding $\mu_{s}\left\{\sigma_{t}\left\{CSA_{\mathrm{rX}}\right\}\right\}$ and $\mu_{s}\left\{{\mathrm{COV}}_{t}\left\{CSA_{\mathrm{rX}}\right\}\right\}$.

The inter-subject variability is represented by the standard deviation across the mean CSA: $\sigma_{s}\left\{\mu_{t}\left\{CSA_{\mathrm{rX}}\right\}\right\}$ and its associated COV: ${\mathrm{COV}}_{s}\left\{\mu_{t}\left\{CSA_{\mathrm{rX}}\right\}\right\}$.

#### 2.3.1 Between-group minimum sample size

Of interest, the minimum sample size (number of subjects per study arm) necessary to detect an atrophy between unpaired study arms was computed based on a two-sample (unpaired) bilateral *t*-test using the following formula (Wang and Ji, 2020, Wittes, 2002):

$$n_{\mathrm{unpaired}}\;=\;\frac{\;{\left(z_{\alpha /2}\;+\;z_{\beta }\right)}^{2}\left(\sigma_{s}{\left\{\mathrm{CS}A_{\mathrm{rX},\;tY}\right\}}^{2}+\sigma_{s}{\left\{\mathrm{CS}A_{r1,\;tY}\right\}}^{2}\right)}{\Delta_{\mathrm{group}}^{2}}$$

where $n_{\mathrm{unpaired}}$ is the minimum sample size required to differentiate between groups given a power (*β*) and level of significance (α). $z_{\beta }$ corresponds to the power z-score, e.g. 80% power gives β = 0.2 and $z_{\beta }$ = -0.84. $z_{\alpha /2}$ corresponds to the significance level z-score, e.g. 5% level of significance gives α = 0.05 and $z_{\alpha /2}$ = -1.96. $\sigma_{s}\left\{CSA_{\mathrm{rX},tY}\right\}$ and $\sigma_{s}\left\{CSA_{r1,tY}\right\}$ are respectively the inter-subject standard deviation of the rescaled (*rX*) and unscaled (*r1*, native resolution) CSA taken at a random transformation *tY*. $\Delta_{\mathrm{group}}$ is the theoretical difference between the average CSA of each group:

$$\Delta_{\mathrm{group}}=\mu_{s}\left\{\mu_{t}\left\{CSA_{r1}\right\}\right\}\cdot \left(1-rX^{2}\right)$$

#### 2.3.2 Within-subject minimum sample size

The minimum sample size necessary to detect an atrophy in a longitudinal (within subject repeated measures) study was computed based on a two-sample bilateral paired *t*-test using the following formula (Altmann et al., 2009):

$$n_{\mathrm{paired}}=\frac{{\left(z_{\alpha /2}+z_{\beta }\right)}^{2}{\left(\sigma_{\mathrm{diff}}\right)}^{2}}{{\Delta_{\mathrm{group}}}^{2}}$$

where $\sigma_{\mathrm{diff}}$ is the standard deviation of the difference between the unscaled and scaled CSA across subjects:

$$\sigma_{\mathrm{diff}}=\sigma_{s}\left\{CSA_{r1,tY}-CSA_{\mathrm{rX},tZ}\right\}$$

Here, we selected random Monte Carlo samples (transformation) for the rescaled and for the unscaled CSA, which are respectively denoted *tY* and *tZ*. In addition, errors on theoretical CSA measures after rescaling were computed. This allowed us to take a deeper look into the effect of the atrophy simulation on the segmentation and CSA measures. To do so, the error was computed using the following formula:

$$\mathrm{Error}=\frac{{\sum_{1}^{n}(\mu_{t}\left\{CSA_{\mathrm{sI},rX}\right\}-\mu_{t}\left\{CSA_{\mathrm{sI},r1}\right\}\cdot \left(rX\right)}^{2})}{n}$$

where $\mu_{t}\left\{CSA_{\mathrm{sI},rX}\right\}$ is the average CSA across Monte Carlo samples with scaling rX, $\mu_{t}\left\{CSA_{\mathrm{sI},r1}\right\}\cdot {\left(rX\right)}^{2}$ is the average unscaled CSA across Monte Carlo samples, multiplied by the scaling coefficient rX squared to account for area change, and *n* is the number of subjects (in this study n = 260).

## 3 Results

### 3.1 Precision and accuracy of atrophy estimation

**Fig. 3:**
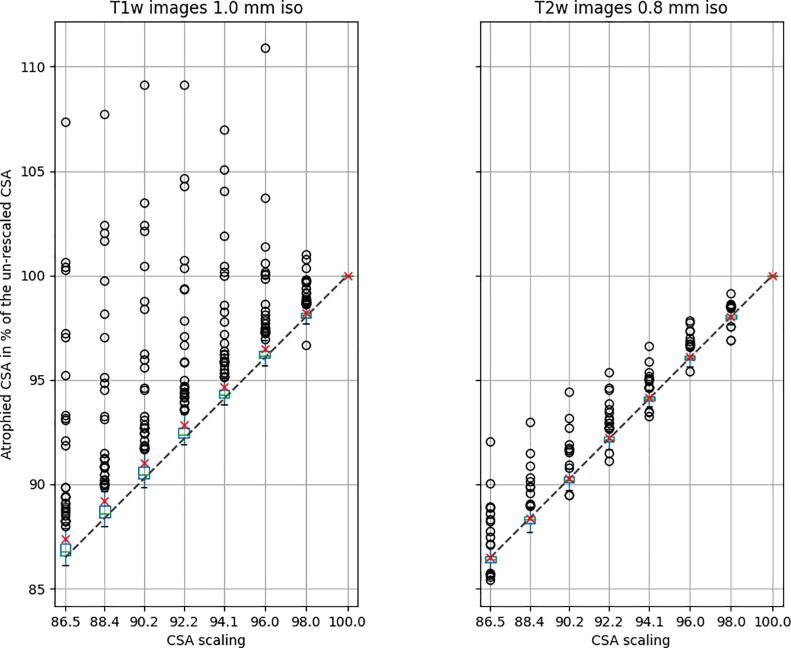
Estimated atrophy as a function of CSA scaling for T1w images with resolution 1.0 mm isotropic (left) and T2w images with resolution 0.8 mm isotropic (right). The green horizontal bar in each boxplot corresponds to the median, the red cross corresponds to the mean, the dotted line represents the ground truth CSA, the boxplot edges represent the interquartile range ($IQR=Q_{3}-Q_{1}$) while the whiskers represent the 1.5×IQR and outliers correspond to the subjects past the whiskers. (For interpretation of the references to colour in this figure legend, the reader is referred to the web version of this article.)

The simulated intra-subject variability (without scaling) expressed with ${\mathrm{COV}}_{t}\left\{CSA_{\mathrm{sI},rX}\right\}$ was 0.8% for T1w images and 0.6% for T2w images. Fig. 3 illustrates the variability of the estimated atrophy as a function of CSA scaling, which is obtained by dividing the estimated CSA at a given scaling factor by the CSA without scaling. This calculation is done independently for every subject, hence there is no variability for the abscissa “100”. The purpose of this figure is to illustrate the variability associated with transformations and scaling, not the inter-subject variability. Overall, the estimated CSA is in agreement with the various degrees of simulated atrophy. We notice a higher number of outliers and variability (i.e., larger quartile bounds) on the T1w vs. on the T2w contrast (discussed in section 4.2).

Table 1 reports the mean absolute CSA error across simulated atrophies. As observed in Fig. 3, error increases as the CSA scaling decreases (i.e., going from right to left on the table). As also observed on the figure, the error is higher on T1w vs. on T2w images.

**Table 1:** Mean absolute CSA error as a function of percent atrophy. “0” corresponds to no atrophy (native resolution).

Atrophy %		**13.51**	**11.64**	**9.75**	**7.84**	**5.91**	**3.96**	**1.99**	**0**
mean error %	T1w images	1.04	0.96	0.85	0.75	0.61	0.44	0.19	0.00
	T2w images	−0.01	0.00	0.02	0.06	0.05	0.06	0.02	0.00

Overall, we observe an underestimation of atrophy (i.e., overestimation of CSA), which amplifies as the simulated atrophy increases (going from right to left on the figure). This underestimated atrophy is larger on the T1w vs. the T2w data. Interestingly, most outliers are prone to over-segmentation rather than under-segmentation (discussed in section 4.1).

### 3.2 Inter-subject variability of CSA

**Fig. 4:**
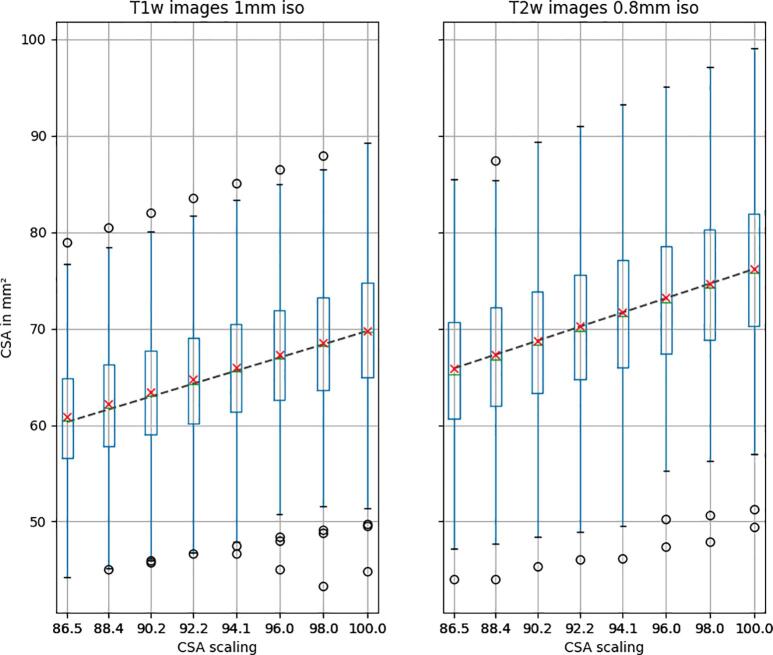
Inter-subject CSA as a function of CSA scaling. The green horizontal bar in each boxplot corresponds to the median, the red cross corresponds to the mean, the boxplot edges represent to the inter-quartile range ($IQR=Q_{3}-Q_{1}$) while the whiskers represent the 1.5×IQR and outliers correspond to the subjects past the whiskers. The black dashed line represents the ground truth CSA. (For interpretation of the references to colour in this figure legend, the reader is referred to the web version of this article.)

The inter-subject variability was computed by calculating the inter-subject mean and standard deviation (denoted $\sigma_{s}\left\{\mu_{t}\left\{CSA_{\mathrm{rX}}\right\}\right\}$ in section 2.3) of the intra-subject mean CSA across Monte Carlo samples (i.e. rigid transformations). Fig. 4 illustrates the dispersion of CSA means across subjects. Overall, there is a good agreement between the mean CSA and the ground truth CSA. We also notice a fairly large inter-subject variability, which is expected as spinal cord sizes vary across people (Papinutto et al., 2020) and no normalization was applied here. Overall, dispersion decreases as the CSA scaling decreases (going from right to left on the figure, 100% being the “unscaled” CSA). This justifies the use of COV as the principal indicator for inter-subject variability, because the reduction of the dispersion is likely associated with the reduction of the mean CSA. We also notice an overall higher CSA estimation on the T2w vs. on the T1w contrast. On the native (unscaled) images, this difference is 6.42 mm^2^. This observation is further discussed in section 4.2.

Table 2 shows the inter-subject COV (defined as ${\mathrm{COV}}_{s}\left\{\mu_{t}\left\{CSA_{\mathrm{rX}}\right\}\right\}$ in *section 2.3*), on the CSA measures for T1w and T2w images, and for each percent atrophy. Overall, the inter-subject COV is similar between the two contrasts, and slightly decreases as the atrophy increases (from right to left).

**Table 2:** Inter-subject COV of CSA across subjects as a function of percent atrophy. “0″ corresponds to no atrophy (native resolution).

atrophy %		**13.51**	**11.64**	**9.75**	**7.84**	**5.91**	**3.96**	**1.99**	**0**
COV inter-subject %atrophy %	T1w images	10.38	10.40	10.43	10.46	10.53	10.62	10.83	10.89
	T2w images	10.85	10.87	10.87	10.86	10.89	10.90	10.94	10.94

### 3.3 Sample size calculation

**Fig. 5:**
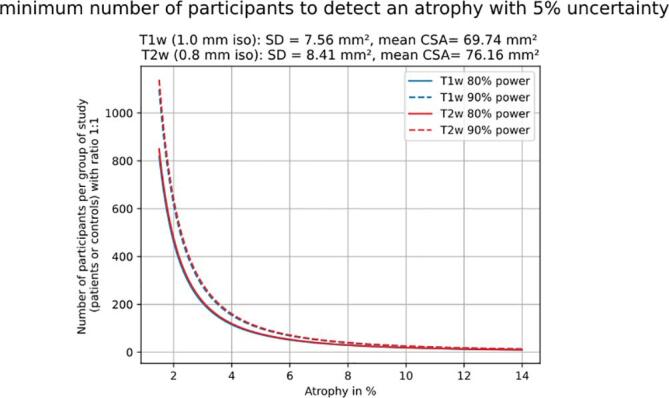
Minimum number of participants required to detect an atrophy. This power analysis is based on a two-sample bilateral *t*-test, with the ratio of patients to controls being 1:1 and a 5% type-I error rate. This analysis was run for T1w (blue) and T2w (red), for 80% (continuous line) and 90% (dashed line) powers. (For interpretation of the references to colour in this figure legend, the reader is referred to the web version of this article.)

Sample size was computed for both cross-sectional and longitudinal studies respectively using the formulas presented in section 2.3.1 and 2.3.2. Fig. 5 represents the minimum sample size required to detect a significant atrophy between unpaired study arms. This figure is consistent with the trends presented in Table 2 (inter-subject COV) demonstrating similar required sample sizes for both contrasts.

Table 3 shows the minimum sample size required to detect a significant atrophy between unpaired study arms for T1w and T2w images, and for each CSA scaling. Note that to simulate “true” conditions, only one Monte Carlo sample (transformation) for each subject was used to compute sample sizes. Then 500 iterations of this Monte Carlo simulation were averaged and variability was estimated.

**Table 3:** Minimum sample size needed for a given atrophy. This power analysis is based on a two-sample bilateral *t*-test, with the ratio of patients to controls being 1:1 and at a 5% type-I error rate.

atrophy %		13.51	11.64	9.75	7.84	5.91	3.96	1.99
Sample size 80% power	T1w images	9 +/− 0.2	12 +/− 0.3	18 +/− 0.4	28 +/− 0.7	50 +/− 1.3	114 +/− 3.2	467 +/− 13.9
	T2w images	9 +/− 0.1	13 +/− 0	18 +/− 0.1	29 +/− 0.2	51 +/− 0.3	116 +/− 0.7	467 +/− 3.2
Sample size 90% power	T1w images	12 +/− 0.3	17 +/− 0.4	24 +/− 0.6	37 +/− 0.9	67 +/− 1.7	152 +/− 4.2	625 +/− 18.6
	T2w images	12 +/− 0.1	17 +/− 0.1	24 +/− 0.2	38 +/− 0.2	68 +/− 0.4	155 +/− 1	625 +/− 4.3

**Fig. 6:**
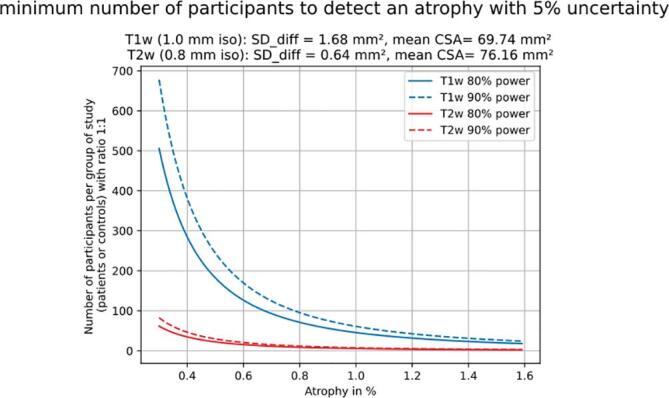
Minimum number of participants required to detect an atrophy. This power analysis is based on a two-sample bilateral *t*-test, with the ratio of patients to controls being 1:1 and a 5% type-I error rate. This analysis was run for T1w (blue) and T2w (red), for 80% (continuous line) and 90% (dashed line) powers. (For interpretation of the references to colour in this figure legend, the reader is referred to the web version of this article.)

Fig. 6 represents the minimum sample size required to detect a significant atrophy in a longitudinal study. This figure is consistent with the trends presented in Fig. 3 (intra-subject variability between scalings) demonstrating a larger required sample sizes for T1w vs. T2w images.

Table 4 shows the minimum required sample size required to detect a significant atrophy in a within subject study for T1w and T2w images, and for each CSA scaling. Note that to simulate “true” conditions only one Monte Carlo sample (transformation) for each subject was used to compute sample sizes. Then, 500 iterations of this Monte Carlo simulation were averaged and variability was estimated.

**Table 4:** Minimum sample size needed for a given atrophy. This power analysis is based on a paired bilateral *t*-test, with the ratio of patients to controls being 1:1 and at a 5% type-I error rate.

atrophy %		1.59	1.20	0.80	0.40
Sample size 80% power	T1w images	14 +/− 5.7	27 +/− 10.3	60 +/− 25.1	229 +/− 90.3
	T2w images	3 +/− 0.3	5 +/− 0.4	10 +/− 1.2	37 +/− 4.9
Sample size 90% power	T1w images	19 +/− 7.7	35 +/− 13.8	80 +/− 33.7	307 +/− 120.9
	T2w images	4 +/− 0.4	6 +/− 0.6	13 +/− 1.6	50 +/− 6.6

## 4 Discussion

The purpose of this article was to gain insights on the minimally detectable SC atrophy using a fully automated pipeline for SC segmentation and vertebral labeling. To promote transparency and reproducibility an open-access data was used (Cohen-Adad et al., 2021), and the analysis code is open-source and fully documented (https://csa-atrophy.readthedocs.io). The method used for simulating atrophy was a global image scaling, while the method used to mimic scan-rescan variability was rigid transformations. An important outcome of this investigation is that a mean atrophy difference of 2% between unpaired study arms, commonly seen in MS (Casserly et al., 2018), could be detected with a minimum of 467 +/− 13.9 subjects using T1w (1 mm iso resolution) and 467 +/− 3.2 subjects using T2w images (0.8 mm iso resolution). Whereas in a longitudinal study, the minimum sample size to detect a 0.4% atrophy between two time points was 229 +/− 90.3 subjects using T1w and 37 +/− 4.9 subjects using T2w images. The discussion below addresses the main findings, limitations and perspectives. We stress that these results correspond to a “best case” scenario, in that the dataset used here was of particularly good quality and the model for simulating atrophy does not encompass all the variability met in real-life datasets.

### 4.1 Inter- and intra-subject variability of CSA estimation

A strength of this study lies in the multi-center dataset used, featuring 260 subjects from 42 international centres (America, Europe, Asia, Oceania), spanning three vendors (GE, Philips and Siemens) and multiple models and software versions. As implied by the geographic diversity of scanning centers, the data includes heterogeneity of ethnic background. The scan quality also varied across centres, due to the level of expertise of the operator, and the subjects themselves. Subjects with notable artifacts are listed in the GitHub repository of the dataset.^3^^,^^4^

The inter-subject SD (COV) of CSA estimation were 7.56 mm^2^ (10.86%) for T1w images and 8.41 mm^2^ (10.90%) for T2w images, which is consistent with previous studies. Weeda et al. reported an inter-subject SD CSA of 4.51 mm^2^ (8.45%) using the SCT-propseg method, 8.22 mm^2^ (15.00%) using the SCT-deepseg method, 10.20 mm^2^ (13.4%) using NeuroQLab, 10.96 mm^2^ (14.54%) using XinapseJIM and 8.49 mm^2^ (11.45%) using ITK-SNAP (Weeda et al., 2019)*.* Yiannakas et al. reported an inter-subject SD CSA of 7.1 mm^2^ (9.81%) using SCT-propseg and 7.4 mm^2^ (9.4%) using XinapseJIM (Yiannakas et al., 2016).

At the intra-subject level, the estimated CSA showed good precision, with COVs (across Monte Carlo transformations) of 0.8% for T1w images and 0.57% for T2w images. When comparing with a previous single-subject scan-rescan study across 19 sites (Cohen-Adad, 2020), scan-rescan COVs on T1w (T2w in brackets) images were respectively 2.3% (2.3%), 1.8% (2.1%) and 0.9% (1.5%) for GE, Philips and Siemens scanners. A notable difference is that, in the spine-generic study, the same subject was scanned across different sites, hence the variability also included possible site-specific differences (scanner, positioning, coil loading, etc.). Conversely, in the present study, intra-subject variability only resulted from rigid transformations. In future work one could improve the realism of the scan-rescan variability by simulating image artifacts and noise (Camara-Rey et al., 2006, Graham et al., 2016).

We noticed several subjects with overestimated CSA. These outliers are visible outside the boxplot whiskers in Fig. 3 particularly on T1w images. Interestingly, the same subjects seemed to be outliers across the different scaling values. A deeper look at these subjects' images did not suggest evident cause for them being outliers. Notably, the following artifacts were looked for: subject motion; cord pulsatile motion; poor shimming; poor fat saturation; aliasing; ghosting; and Gibbs ringing, but none of them were clearly discernible on the outlier subjects. T2w images exhibited less outliers, which could be due to the “cleaner” aspect of the images (i.e. less sensitive to patient motion, sharper SC/CSF border and a higher contrast) between the SC and the CSF and also to better spatial resolution (0.8 mm vs. 1 mm isotropic for the T1w data) which is further investigated in the supplementary material Figure S4 and **Table S4**. The contrast-dependent differences are further discussed in section 4.2. Beyond the visual inspection of image quality to explain these outliers, we also investigated if the precision of CSA estimation across Monte Carlo samples (rigid transformations) had an impact on CSA error. As detailed in Figure S1 and **Table S2**, there is an association between the precision and the accuracy of CSA estimation. Further investigation, detailed in Figure S2, suggests no particular association subject-wise. For example, subjects that are outliers in T1w are not necessarily outliers in T2w contrasts.

### 4.2 Accuracy of CSA estimation and impact of image contrast

As scaling increased, CSA estimation error also increased (Fig. 3). This scaling-dependent bias may be explained by an increase in partial volume effect with tissues outside of the parenchyma which had similar intensity as the cord (e.g. epidural space, ligaments). As the image is further scaled down, the mixture of different tissues in voxels at the SC/CSF interface increases, causing a “leaking” of the segmentation and an overestimation of the CSA. This overestimation is possibly related to the segmentation algorithm, which expects a cord and surrounding tissues to be of a certain dimension. However, the deep learning segmentation approach used here should in principle be less sensitive to these rescalings, because the model training included image scaling during data augmentation. A possible association between CSA size and error on CSA estimation is further investigated in supplementary material Figure S3 and **Table S3**, but no significant association was found. Moreover, during the development of the pipeline, we noticed that the use of different interpolation orders had a small impact on the accuracy of the estimated CSA, but it did not affect the precision.

CSA computed on the T2w images is on average 6.42 mm^2^ larger than that on T1w images. Other studies have reported similar outcomes (De Leener et al., 2014). There are multiple factors that could explain differences: (i) inherent image contrast differences, caused by the fact that tissues don’t have the same relaxation parameters (e.g. the pial matter has a short T2), hence the visible boundary at tissue interface could be slightly shifted. (ii) Image processing, such as the application of a smoothing kernel (apodization), image artifacts including Gibbs ringing, sensitivity to motion and flow artifacts. (iii) Sensitivity of the segmentation algorithm to the CSF/SC contrast difference. Most segmentation algorithms, such as PropSeg (De Leener et al., 2014) and Xinapse JIM (Horsfield et al., 2010) are driven by the image gradient at the tissue interface. Thus, it is not surprising that two different image contrasts yield a different definition of the interface boundary from the segmentation algorithm. Consequently, sct_deepseg_sc (Gros et al., 2019) which was trained from masks generated by PropSeg, then manually corrected, featured the same bias. It is important to note that a systematic bias across software is not an issue when it comes to using CSA values for clinical studies: it only adds an offset and does not affect the precision of the measure. It is similar to a calibration problem. (iv) The native spatial resolution is different between T1w (1 mm iso) and T2w (0.8 mm iso) images. To further investigate the impact of spatial resolution on the accuracy of CSA estimation, T2w images were downsampled to the native resolution of the T1w data (1 mm iso) and also upsampled to 0.5 mm iso. Results of this investigation show that a different spatial resolution affects the association between CSA error and atrophy (Figure S4 and **Table S4**). These results suggest that differences in native image resolutions could partly explain the CSA difference observed between the T1w and T2w contrasts.

### 4.3 Minimum sample size to detect atrophy

Sample size calculation provides an estimation of the minimum number of subjects required to detect a given atrophy between study arms. Even though the observed mean CSA was larger on T2w images than on T1w images (see section 4.2), Table 3 shows that the required number of subjects, to detect a given atrophy, were similar between T1w and T2w contrasts. For example, to detect a 2% atrophy between unpaired study arms, 467 +/− 13.9 and 467 +/− 3.2 subjects are required for T1w and T2w data, respectively. In comparison, the recent paper by Papinutto et al. (Papinutto et al., 2020) reported an inter-subject standard deviation of the CSA of 7.59 mm^2^, and to detect an atrophy of 10% (corresponding to 7.77 mm^2^ in their study) they estimated a minimum sample size of 43 subjects. In our study, we also report an inter-subject standard deviation of 7.59 mm^2^ (the matching number at the 100th decimal is a pure coincidence), and to detect the same atrophy of 10% (corresponding to 6.97 mm^2^) the minimum sample size computed from the formula presented in *section 2.3.1* is 50 subjects (25 subjects per study arm), which is in the same order as the study of Papinutto et al.

Results of sample sizes computed to detect an atrophy between paired study arms were much higher using T1w (229 +/− 90.3) vs T2w (37 +/− 4.9) images. This discrepancy is coherent with the higher intra-subject variability between scalings for T1w vs. T2w images presented in Fig. 3. These sample size results are in the same order of magnitude as the study presented by Altmann et al. (Altmann et al., 2009) for a real clinical longitudinal atrophy study. In the brain, to detect 50% treatment effect (equivalent for progressive MS in the SC atrophy to 1.02%/year (Casserly et al., 2018)) the necessary sample sizes were (respectively for 12, 24 and 36 months) 98, 70 and 60 using CCV power and 47, 28 and 30 using SIENA power.

Looking at the broader picture, even though the required sample size is often larger in comparison with clinical trials using brain atrophy (−1.78% vs − 0.5% per year) (Moccia, Ruggieri, et al., 2019), SC atrophy is increasingly used as an outcome measure (Moccia et al., 2017)*.*

### 4.4 Realism of the atrophy model

The convenience of the highly controlled “global scaling” atrophy model may over-simplify the biological reality. Atrophy models have been studied in the brain using several approaches to “mimic” atrophy and produce ground truth data with known brain volume changes. These studies simulate longitudinal deformation and atrophy for the production of brain ground truth MRI images by introducing various atrophy models based on: (i) known biomechanical brain tissue atrophy values (Camara et al., 2006, da Silva et al., 2020, Khanal et al., 2017); (ii) algorithms modelling target images of atrophied brains (Karaçali and Davatzikos, 2006, Modat et al., 2014); and (iii) CNN and segmentation priors (Bernal et al., 2021).

The present study has more similarities with the method presented by Boyes et al. (Boyes et al., 2006) where ground truth was produced using a global image scaling. Although this method is easy to exploit, it is inherently limited. Firstly, the relative scaling between the structures present in an image is not accounted for. In a realistic atrophy scenario, the SC volume decreases, but not the surrounding bones and muscles. In a global scaling, as in our study, all tissue volumes decrease equally. Secondly, a highly pathological cord likely includes abnormal signals in the image, such as hyper/hypointense lesions. Their presence in the SC could impact the performance of the segmentation algorithm, which in turn could impact the accuracy of CSA estimations. DeepSeg’s deep learning model was trained using data presenting various pathologies (MS, ALS, NMO, degenerative cervical myelopathy, etc.) (Gros et al., 2019) and therefore mitigates bias due to abnormal SCs.

On a broader scale, the direct correlation of axonal loss and atrophy is still debated. Poor correlation has been reported showing that SC CSA underestimates the degree of axonal loss and that the CSA measure should be associated with other histopathological markers such as microstructural abnormalities and axon density (Filippi et al., 2020).

### 4.5 Limitations of binary segmentation

The problem with binary segmentations is the loss of precision. When initially introduced in the 90s, SC CSA measures were performed over a single, or very few, slices. Considering a spatial resolution of 1 mm in-plane, a *true* SC CSA of 70 mm^2^ would be highly sensitive to the inclusion/exclusion of a pixel at that resolution. It would represent a fraction of 1/70 of the total pixel count used to calculate CSA. This number is on the same order of magnitude as the CSA atrophy over a year in MS, which is about 1.78% (Casserly et al., 2018). However, partial volume averaging, an approach introduced in later years, recommended to compute CSA over a larger coverage, e.g. C2/C3, which corresponds to 40 slices (assuming 0.8 mm slice thickness). In that case the pixel precision fraction now represents 1/2800. In the present study, the lack of precision caused by binary masks is therefore mitigated because we compute CSA over a large SI coverage (i.e. C3-C5) as shown in supplementary material **Table S5**.

Another promising workaround is to replace binary segmentation with “soft” segmentation methods, wherein the prediction encodes partial volume information. For example, a segmentation mask with a voxel of value 0.2 would mean that the SC accounts for 20% of the voxel. This approach would produce more precise CSA estimations by minimizing the impact of PVEs. SoftSeg, a recent deep learning framework introduced by Gros *et al*. (Gros et al., 2021), is aiming in that direction by outputting a soft (float values between 0 and 1) instead of a binary segmentation. For example, this method demonstrates better precision for the morphometric analysis of SC gray matter, MS lesions and brain tumor segmentations. Further studies could adapt SoftSeg for segmenting the SC and evaluate if these “soft” segmentations provide better sample size calculations than those obtained here.

## 5 Conclusion

In this study we evaluated the robustness and the sensitivity of an automated analysis pipeline for computing SC cross-sectional area at levels C3-C5. Using simulated SC atrophy (global image scaling) and scan-rescan variability (rigid transformations), we computed the minimum sample size to detect an atrophy between groups (cross-sectional study) or within subjects (longitudinal study). While the realism of the atrophy and scan-rescan variability is limited, the present study benefits from a representative pool of data from 42 different sites worldwide, suggesting that the presented results can be generalized outside of a “single site”. The proposed framework is open-source (https://csa-atrophy.readthedocs.io) and could be re-used to assess the sensitivity of other published methods. It would notably be interesting to assess the performance of the recent Generalized Boundary Shift Integral (GBSI) method, which has been shown to improve sample size for similar datasets (Moccia et al., 2020).

## CRediT authorship contribution statement

**Paul Bautin:** Conceptualization, Data curation, Formal analysis, Investigation, Methodology, Software, Validation, Visualization, Writing - original draft. **Julien Cohen-Adad:** Conceptualization, Data curation, Funding acquisition, Investigation, Methodology, Project administration, Resources, Software, Supervision, Validation, Visualization, Writing - original draft, Writing - review & editing.

## Declaration of Competing Interest

The authors declare that they have no known competing financial interests or personal relationships that could have appeared to influence the work reported in this paper.

## Footnotes

1 https://github.com/spine-generic/data-multi-subject/releases/tag/r20201130

2 https://spine-generic.readthedocs.io/en/latest/analysis-pipeline.html#segmentation-and-vertebral-labeling

3 T1w: https://github.com/spine-generic/data-multi-subject/issues/30

4 T2w: https://github.com/spine-generic/data-multi-subject/issues/39
