# Supplementary material for: Minimum detectable spinal cord atrophy with automatic segmentation: Investigations using an open-access dataset of healthy participants
Source: Neuroimage Clin. 2021 Oct 4;32:102849. doi: 10.1016/j.nicl.2021.102849 (PMC8503570; doi:10.1016/j.nicl.2021.102849)
Supplement: Supplementary data 1 [file mmc1.docx]

**[Table S1](#tbl_atrophy_rate)** presents typical rates of annual spinal cord atrophy for different pathologies.

**[Table S1.](#tbles_atrophy_rate)**Typical rates of spinal cord atrophy for different pathologies

| **Pathology** | MS | ALS | ALS | ALS | NMO |
| --- | --- | --- | --- | --- | --- |
| **Source** | [(Courtney Casserly et al., 2018)](https://paperpile.com/c/FWvhYs/QHu5) | [(Wimmer et al., 2020)](https://paperpile.com/c/FWvhYs/DQqoX) | [(de Albuquerque et al., 2017)](https://paperpile.com/c/FWvhYs/6voWN) | [(Agosta et al., 2009)](https://paperpile.com/c/FWvhYs/JbmXG) | [(Liu et al., 2017)](https://paperpile.com/c/FWvhYs/zQCmb) |
| **annual atrophy rate** | 1.78%/year | 3.4%/year | 6%/year | 3.18%/year | 1.53%/year |

[**Figure S1**](#fis_cov) shows the COV across Monte Carlo transformations (i.e. intra-subject variability) as a function of the mean CSA error (in percentage). The plots are color-clustered per scaling factor. The purpose of this figure is to explore the possible cause for the presence of outliers in [**Figure 3**](#fig_atrophy), especially for the T1w contrast. The associated Pearson’s coefficients are listed in [**Table S2**](#tbl_pearson_cov). All of them are significant (p<0.05 non-corrected, p_corr<0.004 with Bonferroni correction), with an exception for the 1% atrophy case on the T2w image (p=0.009). This suggests an association between the precision and the accuracy of CSA estimation.


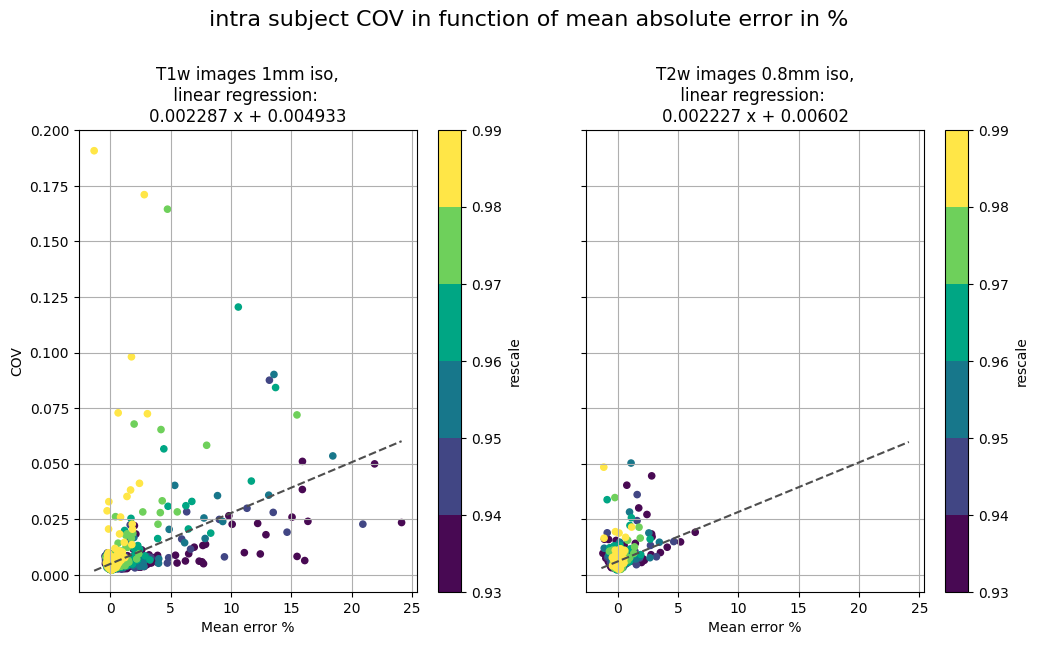


**[Figure S1.](#fisup_cov)** Variability of intra-subject CSA as a function of mean error in % for T1w images (left) and T2w images (right). The colorbar represents the CSA scaling and the dotted line shows the linear regression between mean error and COV

**[Table S2.](#tbles_pearson_cov)** Pearson’s correlation coefficient between intra subject variability of CSA and mean error in %

| atrophy % |  | **13.51** | **11.64** | **9.75** | **7.84** | **5.91** | **3.96** | **1.99** |
| --- | --- | --- | --- | --- | --- | --- | --- | --- |
| pearson's r COV | T1w images | 0.67 | 0.80 | 0.68 | 0.84 | 0.83 | 0.68 | 0.38 |
|  | T2w images | 0.49 | 0.35 | 0.37 | 0.36 | 0.21 | 0.33 | -0.16 |

[**Figure S2**](#fis_cov2) shows the COV across Monte Carlo transformations (ie: intra-subject variability) as a function of the mean CSA error (in percent). The plots are color-clustered per subject, with the same color code between T1w and T2w contrasts. The purpose of this figure, in comparison with [**Figure S1**](#fis_cov), is to check whether subjects that are outliers in T1w are also outliers in T2w contrasts. Visual inspection of this figure suggests no particular association subject-wise.


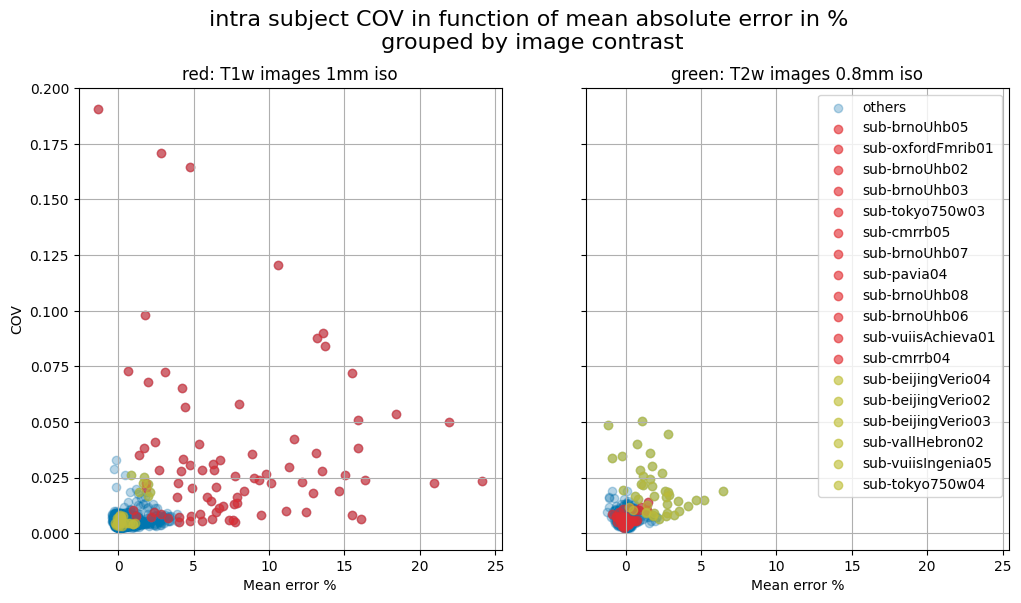


**[Figure S2.](#fisup_cov2)** Intra-subject variability as a function of mean error for T1w images (left) and T2w images (right). Subjects in red are the worst outliers (outside the interval $[Q1-10\times IQR, Q3+10\times IQR]$ percentage error) found in T1w images and subjects in green are the worst outliers found in T2-images while subjects in blue are the rest of subjects (non-outliers).

The presence of outliers in [**Figure 3**](#fig_atrophy), and an increase in CSA overestimation when images were scaled down, lead to the hypothesis that smaller CSAs (or atrophied CSAs) could increase the unreliability of segmentation (or be less sensitive to atrophy on smaller CSAs) and thereby result in a higher COV. [**Figure S3**](#fis_csa) shows the CSA as a function of mean error in %. Note that the CSA were normalized (dividing mean CSA by 'CSA scaling') allowing direct comparison of mean CSA across scalings. Ideally, without any biases (increase in error) for small CSA, the dotted line should be horizontal.

The associated Pearson’s coefficients are listed in [**Table S3**](#tbl_pearson_csa). All of them are significant (p<0.05 non-corrected). However, only 4% scaling for T2w images and 2, 3 and 4% for T1w images are significant with Bonferroni correction (p_corr<0.004). This suggests that there is no direct association between the CSA size and the accuracy of CSA estimation.


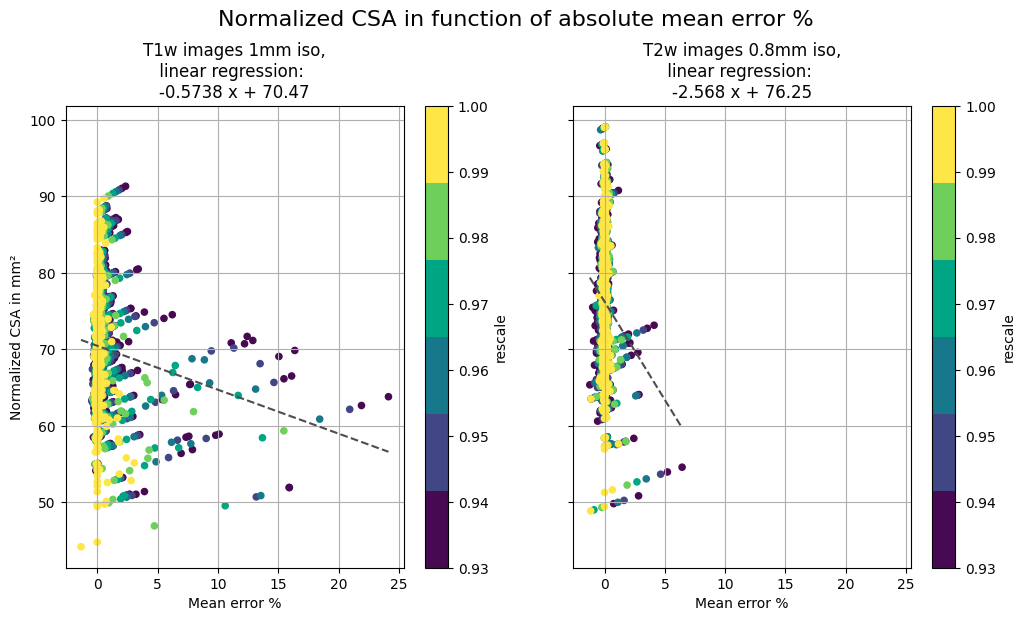


**[Figure S3.](#fisup_csa)** Normalized CSA as a function of mean error in % for T1w images (left) and T2w images (right). The colorbar represents the CSA scaling and normalization applied to each subject’s CSA, the dotted line shows the linear trend of normalized CSA in function of mean error.

**[Table S3.](#tbles_pearson_csa)** Pearson’s correlation coefficient between normalized CSA and error in %

| **atrophy %** |  | **13.51** | **11.64** | **9.75** | **7.84** | **5.91** | **3.96** | **1.99** |
| --- | --- | --- | --- | --- | --- | --- | --- | --- |
| pearson's r CSA | T1w images | -1.44E-01 | -1.57E-01 | -1.72E-01 | -2.07E-01 | -2.34E-01 | -2.24E-01 | -1.69E-01 |
|  | T2w images | -1.64E-01 | -1.45E-01 | -1.62E-01 | -2.13E-01 | -1.63E-01 | -1.54E-01 | 2.18E-02 |

Notable differences between the CSA estimation of T1w and T2w images could be explained by the difference in the native spatial resolution (1 mm iso for the T1w vs. 0.8 mm for the T2w data). [**Figure S4**](#fis_atrophy) shows the intra-subject variability of CSA as a function of CSA scaling for 1 mm, 0.8 mm (native) and 0.5mm isotropic, and [**Table S4**](#tbl_resolution) shows the mean absolute CSA error as a function of percent atrophy, across image resolutions. Results demonstrate that the CSA accuracy is affected by the image resolution, which could partly explain the discrepancies in CSA accuracy observed between the T1w and the T2w results.

**[Figure S4.](#fisup_atrophy)** Variability of the estimated atrophy as a function of CSA scaling for T2w images downsampled at 1.0 mm isotropic (left), at the native 0.8 mm isotropic resolution (center) and upsampled at 0.5 mm isotropic (right). The green horizontal bar in each boxplot corresponds to the median, the red cross corresponds to the mean, the dotted line represents the ground truth CSA, the box corresponds to the inter-quartile range ($IQR=Q_{3}-Q_{1}$) while the whiskers correspond to the $1.5\times IQR$ and outliers correspond to the subjects past the whiskers.
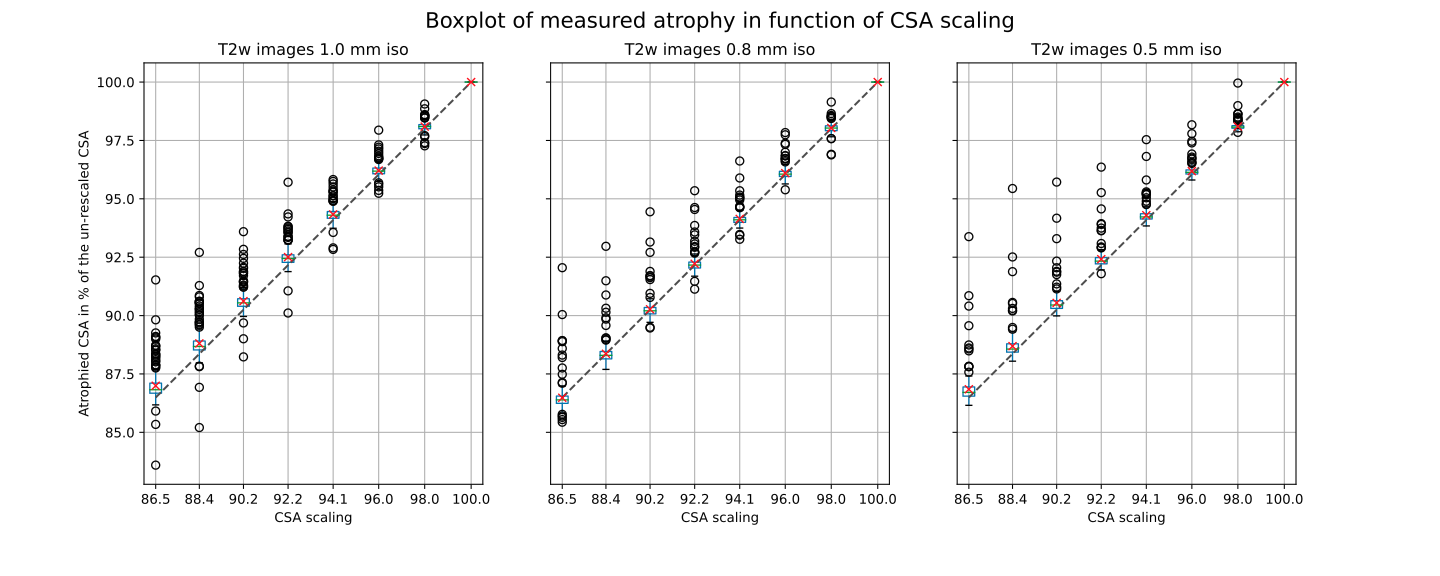


**[Table S4.](#tbles_resolution)** Mean absolute CSA error as a function of percent atrophy. “0” corresponds to no atrophy (native resolution). Here we notice an effect of the resolution on the accuracy of CSA.

| **atrophy %** |  | **13.51** | **11.64** | **9.75** | **7.84** | **5.91** | **3.96** | **1.99** | **0** |
| --- | --- | --- | --- | --- | --- | --- | --- | --- | --- |
| mean error % | T1w images at 1mm | 1.04 | 0.96 | 0.85 | 0.75 | 0.61 | 0.44 | 0.19 | 0.00 |
|  | T2w images at 0.8mm | -0.01 | 0.00 | 0.02 | 0.06 | 0.05 | 0.06 | 0.02 | 0.00 |
|  | T2w images at 1mm | 0.36 | 0.32 | 0.26 | 0.25 | 0.17 | 0.12 | 0.06 | 0 |

Intra-subject variability incorporates variability caused by the loss of precision associated with the binary segmentation. It is therefore expected that the more slices (ie: voxels) are used to compute the average CSA, the more precise (ie: lower COV) results will be. [**Table S5**](#tbl_vertebrae) below shows the intra-subject coefficient of variation (COV) as a function of percent atrophy for one, two and three vertebrae. The average number of slices (across participants) is reported for each scenario. As expected, when the number of slices used to compute CSA increases, the COV decreases.

**[Table S5.](#tbles_vertebrae)** Intra-subject coefficient of variation (COV) as a function of percent atrophy. “0” corresponds to no atrophy (native resolution).

| **atrophy %** |  | **Number of slices** | **13.51** | **11.64** | **9.75** | **7.84** | **5.91** | **3.96** | **1.99** | **0** |
| --- | --- | --- | --- | --- | --- | --- | --- | --- | --- | --- |
| COV intra-subject | vertebrae [3] | 21.7 | 0.010 | 0.010 | 0.010 | 0.009 | 0.009 | 0.009 | 0.009 | 0.009 |
|  | vertebrae [3,4] | 42.2 | 0.008 | 0.008 | 0.007 | 0.007 | 0.007 | 0.007 | 0.007 | 0.007 |
|  | vertebrae [3,4,5] | 61.9 | 0.006 | 0.006 | 0.006 | 0.006 | 0.006 | 0.006 | 0.006 | 0.006 |
